# Using a mHealth system to recall and refer existing clients and refer community members with health concerns to primary healthcare facilities in South Africa: a feasibility study

**DOI:** 10.1080/16549716.2020.1717410

**Published:** 2020-02-10

**Authors:** Willem Odendaal, Simon Lewin, Brian McKinstry, Mark Tomlinson, Esme Jordaan, Mikateko Mazinu, Pam Haig, Anna Thorson, Salla Atkins

**Affiliations:** a Health Systems Research Unit, South African Medical Research Council, Cape Town, South Africa; b Department of Psychiatry, Stellenbosch University, Stellenbosch, South Africa; c Division of Health Services, Norwegian Institute of Public Health, Oslo, Norway; d Usher Institute of Population Health Sciences and Informatics, The University of Edinburgh, Edinburgh, UK; e Department of Global Health, Institute for Life Course Health Research, Stellenbosch University, Stellenbosch, South Africa; f School of Nursing and Midwifery, Queen’s University, Belfast, UK; g Biostatistics Unit, South African Medical Research Council, Cape Town, South Africa; h Statistics and Population Studies, University of the Western Cape, Cape Town, South Africa; i Family South Africa (FAMSA) Karoo, Oudsthoorn, South Africa; j Department of Global Public Health, Karolinska Institutet, Stockholm, Sweden; k New Social Research and Faculty of Social Sciences, Tampere University, Tampere, Finland

**Keywords:** Client referral, community-based services, continuity of care, healthcare facility, lay health workers, mobile health, primary healthcare, recall to care

## Abstract

**Background**: Lay health workers (LHWs) are critical in linking communities and primary healthcare (PHC) facilities. Effective communication between facilities and LHWs is key to this role. We implemented a mobile health (mHealth) system to improve communication and continuity of care for chronically ill clients. The system focused on requests from facility staff to LHWs to follow up clients and LHW referrals of people who needed care at a facility. We implemented the system in two rural and semi-rural sub-districts in South Africa.

**Objective**: To assess the feasibility of the mHealth system in improving continuity of care for clients in PHC in South Africa.

**Method**: We implemented the intervention in 15 PHC facilities. The clerks issued recalls to LHWs using a tablet computer. LHWs used smartphones to receive these requests, communicate with clerks and refer people to a facility. We undertook a mixed-methods evaluation to assess the feasibility of the mHealth system. We analysed recall and referral data using descriptive statistics. We used thematic content analysis to analyse qualitative data from semi-structured interviews with facility staff and a researcher fieldwork journal.

**Results**: Across the sub-districts, 2,204 clients were recalled and 628 (28%) of these recalls were successful. LHWs made 1,085 referrals of which 485 (45%) were successful. The main client group referred and recalled were children under 5 years. Qualitative data showed the impacts of facility conditions and interpersonal relationships on the mHealth system.

**Conclusion**: Using mHealth for recalls and referrals is probably feasible and can improve communication between LHWs and facility staff. However, the low success rates highlight the need to assess facility capacity beforehand and to integrate mHealth with existing health information systems. mHealth may improve communication between LHWs and facility staff, but its success depends on the health system capacity to incorporate these interventions.

## Background

South Africa is a middle-income country with a high degree of economic inequality [1]. The disease burden of the country is characterised by high rates of communicable, non-communicable, maternal and perinatal and injury-related deaths, also unequally distributed within the population [2]. The leading causes of death are communicable diseases (33.6% of all deaths are caused by HIV/AIDS and TB), and non-communicable diseases, such as cerebrovascular and ischaemic heart diseases, diabetes, and hypertension, account for 19.3% of deaths [2]. This epidemiological situation requires a comprehensive response at all levels of the health system, including strengthening district-based primary healthcare (PHC) and developing innovative interventions [3]. Despite many innovations to improve the public health services in South Africa, such as the National Health Insurance scheme [4], and the PHC re-engineering programme [5], substantial challenges in service provision remain. An important challenge in South Africa is the shortage of skilled health professionals [6], which often leaves existing skilled staff overburdened and demotivated [7].

To address this challenge, a task-shifting approach is widely used in South Africa. One component of this is the use of lay health workers (LHWs) to support nurses and other healthcare professionals in implementing primary healthcare (PHC) at the community level [8]. Currently, LHWs play a critical role in extending the PHC system into communities [5]. South Africa has approximately 72,000 LHWs organised in ward-based outreach teams [9]. They perform a variety of tasks, such as medication administration, child health surveys, referring people with illness symptoms to facilities, and recalling clients to for various reasons, for instance, to receive tests results or medication [10]. LHWs are also key to ensuring that clients are not lost to follow-up [10,11]. Despite these important functions being performed by LHWs, their turnover is high [12], resulting in a need for constant retraining of staff [13]. This turnover can partly be influenced by the characteristics of LHWs’ work: long distances between communities and facilities to receive instructions [14–16], or to report on their work [17], that they usually have to travel on foot. This is added to by other issues, including inadequate reimbursement and poor communication between LHWs and facility staff [15].

Mobile health (mHealth) technologies, defined as medical and public healthcare practices supported by mobile devices such as mobile – and smartphones, client-monitoring devices, and tablets [18], are increasingly used in low- and middle-income countries (LMICs) to help solve health system challenges [17,19,20]. The technology has the potential to address some of the issues that LHWs experience, including traveling time, administrative tasks, and communication with facility staff [17,19–21]. There is also potential for these technologies to improve clients’ access to care and potentially reduce LHWs’ workload [17,22]. mHealth interventions can also contribute to improving the continuity of care for chronic conditions [23–26]. Given this potential, we developed and implemented a mHealth system within a rural and semi – rural PHC programme in South Africa. The programme aimed to improve continuity of care for clients, while improving communication between community-based LHWs and health facilities. This paper reports on a formative evaluation of this programme, focusing on the feasibility of implementing a mHealth system to improve the continuity of care for PHC service users. The findings of the evaluation contribute to understanding the feasibility of these programmes and factors that may affect their sustainability.

## Methods

### Aim

To assess the feasibility of a mHealth system to improve the continuity of care for clients in a PHC programme in South Africa.

### Study design

This was a mixed-methods evaluation study, implemented over 10 months, from June 2015 to March 2016. We used both quantitative and qualitative methods of data collection and analyses to assess the feasibility of the multi-component mHealth system [27,28]. This required a research team experienced in both approaches [29]. We used an embedded design in which the primary focus was on the quantitative dataset. We then used the qualitative data to understand and explain the quantitative findings [30].

### Setting

We planned to implement the mHealth system in a rural setting, as we thought that these settings would benefit most from the improved communication offered through a mobile health system. Following consultations, the Western Cape Department of Health suggested that the study be conducted in two rural sub-districts in the Eden Health district (Flowchart 1), one of the seven health districts in the Western Cape Province of South Africa. These two study sub-districts were selected because each already had a well-functioning team of LHWs.

The Eden district has a total estimated population size of 613,124 [31]. The two study sub-districts represented approximately 21% of the total district population and were substantially different in size (the population of Sub-district 1 estimated at 26,064 and Sub-district 2 at 101,298) [32,33]. Most residents’ first language is Afrikaans (73%), followed by isiXhosa (25%) and English (2%) [34]. In 2016, 40.5% of the population in the district lived below the poverty line (USD 320 per month) [31]. The Eden district performs slightly worse on key health indicators, compared to the Provincial average. For example in 2015, 16% of newborns were underweight in the district, compared to the 14.5% average in the Western Cape, and the maternal mortality rate was higher in Eden (69.9/100 000 live births) when compared to the Provincial rate (58.3/100 000) [31].

PHC facilities in the Eden sub-districts provide basic healthcare services, including treatment for TB, HIV/AIDS and non-communicable diseases, and maternal and child health services. The hospital in Sub-district 1 that participated in the study provides maternity services, basic surgery, and emergency services. Clients who needed to consult a doctor had to book an appointment in advance as doctors visited the respective facilities only on certain days of the month.

### Participants

As noted earlier, each of the selected two study sub-districts had a well-functioning team of LHWs (Flowchart 1), and most of these LHWs lived in the communities in they worked. In each of these districts, the Provincial Department of Health had contracted a non-governmental organisation (NGO) to recruit, manage and pay LHWs to provide community-based PHC services on behalf of the Department of Health. This form of contracting out is common in the Western Cape Province [35]. The LHWs received non-professional training on various topics related to the services they provide, which include supporting clients whom the health facility assigns to them as well as health promotion activities. The LHWs worked 4.5 h a week and earned between 99 and 122 US$ per month, depending on their level of training. At the time of the study, the NGO in Sub-district 1 had two LHW teams (29 LHWs in total), and the NGO in Sub-district 2 had four teams (64 LHWs in total). Each team was supported by a supervisor, who was a retired nurse. Demographic data for the LHWs and supervisors within each sub-district is detailed in Table 1.

**Flowchart 1.**
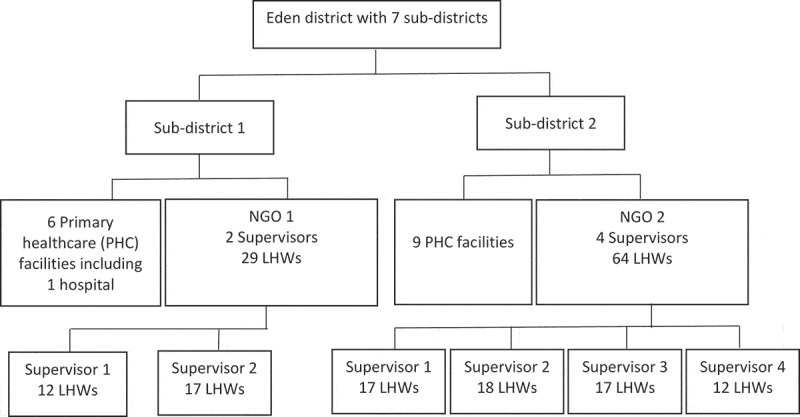
Settings in which the study was implemented

**Table 1.** Supervisor and LHW demographics

	Sub-district 1		Sub-district 2	
	Supervisors	LHWs	Supervisors	LHWs
Female	2	29	4	62
Male	-	-	-	2
Average age	58	35	58	34
Age range	55 – 63	22 – 59	47 – 68	23 – 60
Average number of years working as supervisor or LHW	8	6	3	5
Years in post – range	4 months – 7 years	1 – 10 years	6 – 10 years	3 – 10 years

No demographic data were collected about the clerks.

### The mHealth system

The three key role players involved in the implementation of the mHealth system were the LHWs, their supervisors, and mHealth clerks, henceforth referred to as *clerks*. Each facility manager appointed one staff member, in most cases from the administrative staff, to act as clerk. Managing the mHealth system was in addition to the clerks’ other duties. LHWs were given smartphones for the project, and clerks and nurses tablets, to manage the system.

The main feature of the system was to enable LHWs to record their routine client visits, that is monitoring how they were doing on treatment, do pill counts, and conducting general health assessments in clients’ households, on project-funded smartphones. The system enabled real-time access for supervisors to these reports. The system also enabled two-way communication between LHWs and clerks. LHWs, supervisors and clerks received 2 days of training on how to use the system, offered by a for-profit mHealth service provider (Mobenzi), who developed the system. Thereafter, the implementation staff had 2 weeks to practice using the system before the system went live. Though there were a paper-based recall and referral system in use before the intervention, it was not standardised. The mHealth system could be considered a completely new system to the participating facilities.

**Figure 1.**
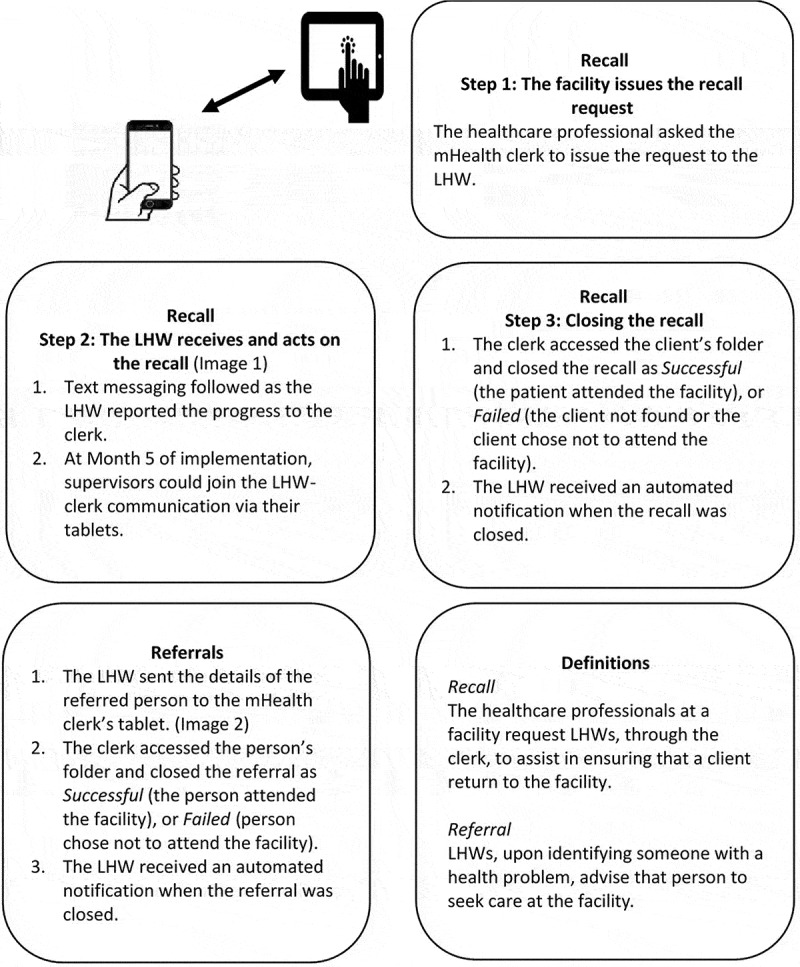
mHealth recall and referral system

**Figure 2.**
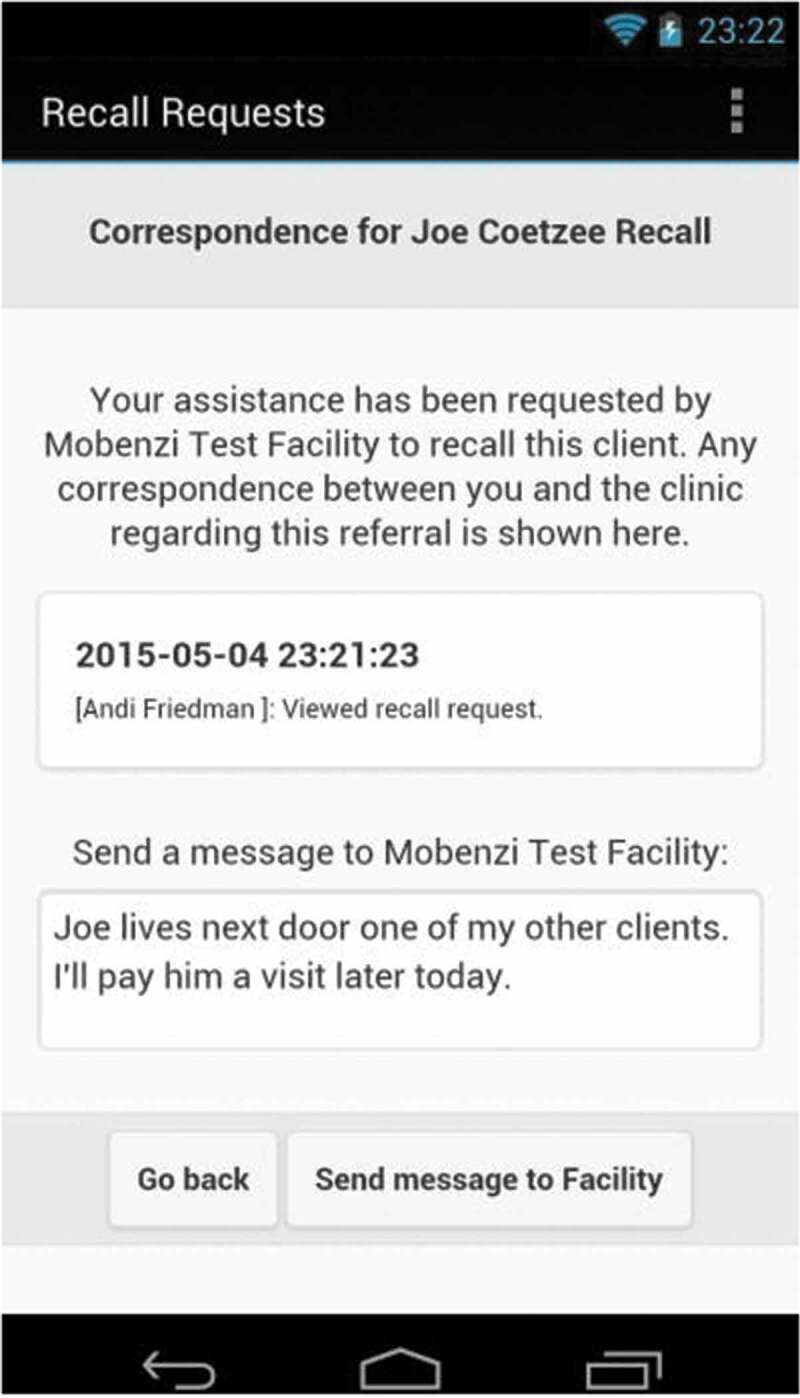
Example of the recall format of correspondence between the LHW and clerkfacility

The mHealth recall and referral process were as follows (Figure 1): Firstly, the healthcare professionals at the facility instructed the clerk to ask an LHW to locate and advise a client to return to the facility (hereto referred to as *recalls*, Figure 2). The clerks issued these requests through the tablet, and these were received by the LHW on their smartphones while working in the community. Real-time communication ensued between clerks and LHWs when they discussed recall progress using the system. The supervisors’ role in the recall process was added in month 5 of the implementation, after this was requested by them. The late addition was due to pre-project consultations suggesting that this functionality was not necessary.

The figure shows pseudonyms for client, LHW and facility, and LHWs in the study were referred to as community care workers (CCWs).

**Figure 3.**
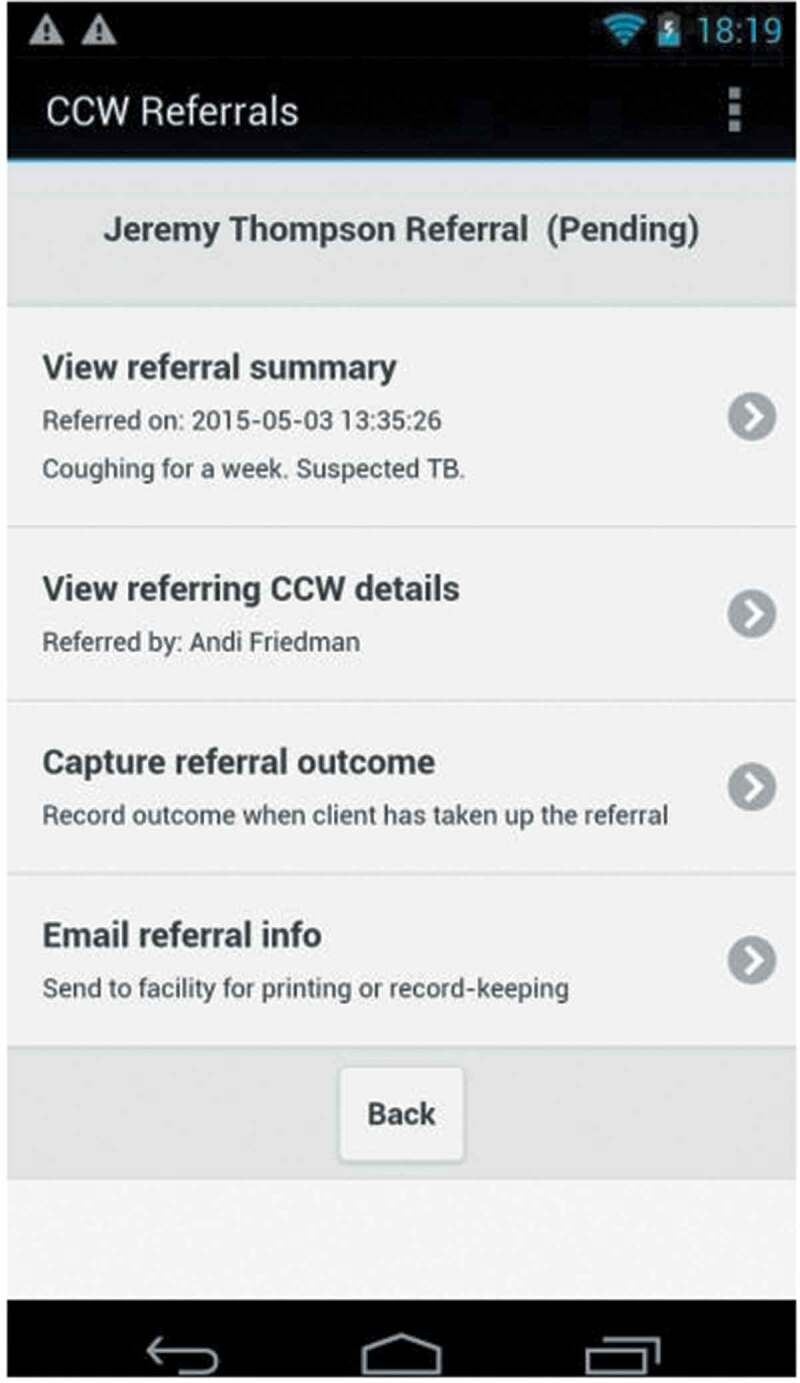
Example of the format for a referral sent by an LHW to the health facility

Secondly, when LHWs identified a person, who could have been an existing client or someone else in the community with a health problem, for example, headaches or wounds requiring care, they would advise that person to seek care at the facility (hereto referred to as *referrals*, Figure 3). LHWs sent a notification of these instances to the clerk’s tablet, using their smartphones. The clerks closed recalls and referrals, respectively, as successful, when the person arrived at the facility, or unsuccessful when the person failed to attend at the facility.

The figure shows pseudonyms for client, LHW and facility, and LHWs in the study were referred to as community care workers (CCWs).

The digital health interventions included in the mHealth system evaluated in this study targeted healthcare providers and can be classified as follows, using the World Health Organization’s classification of digital health interventions [36]:

- *Interventions focused on client health records*: Longitudinal tracking of clients’ health status and services (classification number 2.2.1)
- *Interventions focused on healthcare provider decision support*: Provide checklist according to protocol (classification number 2.3.2)
- *Interventions focused on healthcare provider communication*: Communication from healthcare provider(s) to supervisor (classification number 2.5.1)
- *Interventions focused on referral coordination*: Manage referrals between points of service within health sector (classification number 2.6.2)
- *Interventions focused on health worker activity planning and scheduling*: Schedule healthcare provider’s activities (classification number 2.7.2)

### Data collection

The main question we wanted to answer using quantitative data was whether the mHealth system allowed facility staff and LHWs to, respectively, recall existing clients and refer community members with health concerns to healthcare at the health facilities. All recall and referral data via the mHealth system in the two study sub-districts were stored on a Mobenzi server and exported to Excel by the research team. The data included a date and time stamp, sender and recipient, geographic location, and content of the messages between LHW and clerk. As reasons for recalls and referrals were recorded without predetermined categories, the research team manually coded the recall and referral reasons, categorising them according to most frequent reasons – the codes developed are shown in Table A1. As we collected service indicators that were not in use in the study sites prior to this study, baseline data were not available.

The qualitative component of the study aimed to provide an understanding of the implementation processes and how the participants perceived the mHealth system. These data were intended to help us contextualise the quantitative findings through incorporating participant perspectives. We used two methods of qualitative data collection: semi-structured individual and group interviews, conducted at the end of the project with all of the participating LHWs, supervisors, clerks, and facility managers; and a fieldwork journal kept during the implementation of the study. WO collected the data. The interview questions included how LHWs, supervisors, and clerks felt about using the mHealth system; whether this system changed their practices; their views regarding barriers and facilitators to implementation; and how the mHealth system compared to the paper-based system. We invited the facility managers to join the interviews with the clerks, as it was important for us to ascertain their perceptions, experiences, and recommendations, too. We include the findings from seven clerk/manager interviews, as these cadres were key to the implementation of the mHealth system within the facility, and had a good overall view of the implementation processes. In total, 12 of the 15 clerks, and three of the eight facility managers participated in the interviews. In some instances, facility managers were responsible for two facilities, and the four participating mobile facilities were managed by some of the ‘fixed facility’ managers. The remaining three facilities and clerks were not available at the time that WO conducted these interviews. The staff of some facilities were interviewed together as this was the most convenient approach for gathering data from staff in remote, neighbouring facilities.

The fieldwork journal detailed the researcher’s reflections and observations during the fieldwork visits. For instance, visiting the LHWs in the most remote areas highlighted the challenges of regular contact with, and reporting to, supervisors based in the main towns in the respective sub-districts.

### Analysis

Recalls were categorised as successful if the clerk recorded that the client attended the facility as requested. Failed recalls included the following categories: (i) clients who failed to attend the facility as requested; (ii) unclosed recalls, i.e. where either the clerk or LHW did not respond to the other’s latest correspondence; (iii) LHWs who did not view the recall; and (iv) clerk errors, for example, when the clerk sent the request to the wrong LHW. There were no time limits on keeping recalls open. Referrals were categorised as successful if a person attended the facility for the reason he/she was referred by the LHW. Referrals expired within 14 days of being issued and were then categorised as failed referrals. We collected data from June 2015 until February 2016, the second last month of the study, to ensure that recalls and referrals issued in February could be acted upon by the end of the study in March 2016.

We used Excel and R statistical software (https://www.r-project.org/) for the descriptive statistics, calculating facility- and sub-district level averages of recall and referral numbers and success rates, and to present participant demographics. In order to understand and contextualise our results, we applied a qualitative content analysis approach to the interview data from clerks and facility managers [37]. We used Atlas.ti version 8.1 (https://atlasti.com/product/v8-windows/) to conduct this content analysis. As we were primarily interested in explaining the recall and referral outcomes, we analysed the interview data deductively at the manifest level [37]. We used both the apriori themes from the interview guides, including, e.g. barriers and facilitators to implementation, while allowing additional themes to emerge from the data to explain our quantitative results. SA read and reread the transcripts to familiarise herself with the data and generated condensed meaning units from the data. These meaning units were then further reduced to codes, from which categories were generated that related directly to the quantitative data, see Table 2 for an example of the analysis. The analysis was checked by WO, and differences resolved by discussion. WO referred to the fieldwork journal as the interviews were being analysed, looking for content that could illuminate and further explain the views that participants shared during the interviews. The qualitative and quantitative data are reported in parallel in the results below.

**Table 2.** Example of qualitative analysis

Categories	Sub-categories	Example codes	Examples of extracts from the data
Lack of support and time	Need technical support **Maintaining dual systems** Additional workload After hour recalls	LHWs had difficulties in maintaining two systems	‘*Some of them struggled to use paper and the phone. So I either carry on with the paper or I carry on with the phone … ’*
Communication and interpersonal challenges	**Users struggled adapting to mHealth** LHW-facility staff relationships	LHW: difficulties in the beginning because not familiar with smart phone	*‘like I’ve said, in the beginning when I first went for this training I was nervous. Because I have never used something like this in my life. The only time I might have used a phone, then it was a phone with buttons. But it’s not these modern phones.’*
Closing recalls and referrals	Facility staff communication delay System description **Immediacy**	NC: mH offered immediacy to LHW-facility communication	*‘And when I walk in there the sister would quickly tell me, listen, I’ve passed on this or that to the tablet, or I’ve given this and that to that person. And almost miraculously, when I get to those carers and I’ve already got it from the sister early in the morning, then I get to the CCWs: you know what, I got this and that referral.’*
Effects of mHealth	**Remote settings** Improved communication and reporting Improved recalls **Paper vs mHealth**	NC: very difficult to get data from remote living LHWs	*“ … I got those every second week. I had to arrange it with them because we have to stick to our petrol budget; in other words, I can’t drive to [Place A]* *and [Place B] every week. I was able to go about once a month, and then I had it delivered every second week at the [Place C], that’s the satellite, so I had an arrangement with that man that I would collect it on a Friday.”*

Categories, sub-categories and example codes and quotes (sub-category in bold where codes and quotes are related).

### Ethics, consent and permissions

Prior to conducting this study, ethical approval was obtained from the South African Medical Research Council (EC016-11/2014). The approval included the participant information sheet and informed consent form signed by all participants. All interviewees provided signed informed consent, and the interviews took place in a private space in their respective facilities, at a time that was convenient for them. The interviews were conducted in the language preferred by participants, which was predominantly Afrikaans. Interviews were audio-recorded, transcribed and translated into English. Before the study commenced, we provided the LHWs with an information flyer in plain language and asked them to use this when describing the study to their clients. LHWs were instructed during the training to only use the mHealth system after clients were briefed and had given consent to participate.

## Results

The quantitative results for recalls of PHC clients and referrals of clients and community members are presented below, according to success rates. The categories emerging from the qualitative data – *lack of support and time, communication and interpersonal challenges, closing recalls and referrals*, and the *effects of mHealth –* are reported together with the quantitative data. We first present the recall results and then discuss the referral results.

### Recalls

In total, 2,204 client recalls were issued across the two sub-districts, of which 28% (628/2,204) resulted in clients attending at the facility as requested (Table 3). Most recalls were initiated in Sub-district 2 (1642, 74% of total recalls) as could be expected due to the larger sub-district population. However, the recall success rate in Sub-district 1 was 53% (301/562), compared to 20% (372/1,642) in Sub-district 2. The two most common recall categories across the two sub-districts were recalling children under five (23%, n = 514), for example, for growth or nutrition monitoring or vaccinations, and facility appointment reminders (10%, n = 230), that is reminding clients of upcoming appointments, and in many cases, of rescheduled appointments (see Table A1 for further detail).

**Table 3.** Recall success rates according to the recall reason

Reason for recall		Sub-district 1 N (%)	Sub-district 2 N (%)	Total across sub-districts N (%)^a^
Children < 5 years (e.g. deworming, Vitamin A, immunisation)	Successful recalls	101 (64%)	75 (21%)	176 (34%)
	*Total recalls*	*158*	*356*	*514 (23%)*
Diagnostic tests (being tested or receiving results for all conditions excluding TB/HIV and AIDS)	Successful recalls	22 (44%)	11 (9%)	33 (20%)
	*Total recalls*	*50*	*117*	*167 (8%)*
Finding defaulting clients (including TB/HIV clients)	Successful recalls	4 (44%)	11 (15%)	15 (19%)
	*Total recalls*	*9*	*72*	*81 (4%)*
Medication collection	Successful recalls	16 (55%)	51 (15%)	67 (17%)
	*Total recalls*	*29*	*348*	*377 (17%)*
Non-communicable disease care	Successful recalls	5 (83%)	7 (39%)	12 (50%)
	*Total recalls*	*6*	*18*	*24 (1%)*
Obstetrics/Gynaecology (including family planning)	Successful recalls	4 (27%)	3 (5%)	7 (9%)
	*Total recalls*	*15*	*62*	*77 (3%)*
Reminding clients about facility appointments	Successful recalls	86 (57%)	9 (11%)	95 (41%)
	*Total recalls*	*150*	*80*	*230 (10%)*
TB/HIV/AIDS care	Successful recalls	8 (44%)	10 (16%)	18 (21%)
	*Total recalls*	*18*	*64*	*82 (4%)*
Other^b^	Successful recalls	55 (43%)	150 (29%)	205 (31%)
	*Total recalls*	*127*	*525*	*652 (30%)*
Total	Successful recalls	301 (53%)	327 (20%)	628 (28%)
	*Total recalls*	*562 (26%)*	*1,642 (74%)*	*2,204 (100%)*

^a^The % reported for the total of each recall reason is the proportion of the total recalls across sub-districts.

^b^Included a range of health issues, such as wound care, eye care, having to see the occupational therapist or social worker, and mental healthcare. It also included unspecified reasons, when the recall/referral simply stated that the client needed to seek care at the facility.

Table 3 shows the recall success rates according to the recall reasons. There was a high number of medication collection recalls in Sub-district 2 (21% of the total recalls) because of fewer community-based medication dispensing outlets available for the population than what was available in Sub-district 1. Many Sub-district 2 clients, therefore, had to collect their medication at the nearest health facility and thus became part of the mHealth recall system. The high number of facility appointment reminder recalls in Sub-district 1 (27% of total recalls in that sub-district) were due to frequent doctor appointment rescheduling (personal communication, facility manager, 8 April 2016). Qualitative data suggested that clerks thought that the mHealth system supported this rescheduling well, as it speeded up messages getting to clients via LHWs, adding to the system’s feasibility.

To facilitate ownership of the mHealth system, we decided in advance to allow clerks and LHW teams to adapt the system in any way that made it easier for them to use. The interview data suggested that this happened in relation to the process of issuing recalls, which differed across facilities. In some facilities, recall requests were made through a meeting among the healthcare professionals, while in others, clerks received a stack of folders or were told verbally or through stickers or book notes to recall clients. Facility staff also noted that when the period for facility audits, i.e. reporting on how well the facility performed against service targets, was approaching, certain client categories would be prioritised for recalls, to meet these targets. One clerk noted the following when asked about the improvement in recall numbers:

*Audits! … When an audit is coming, then the patients are called in because we are worried that we won’t get it* [the targets] *right*. (Sub-district 1, Facility 1 clerk)

The qualitative data from over half of the participating facilities indicated that interpersonal relationships and communication patterns in the facility and with LHWs could impact substantively on closing recalls, and thus impacted on the feasibility of the mHealth system. As reported below, interpersonal conflicts meant that information about clients arriving was not communicated, hampering system implementation:

*The communication between us* [clerks and healthcare professionals] *was just not right to say that the patient did come back….*(Sub-district 2, Facility 1 clerk)

### Referrals

A total of 1,085 referrals were recorded across the two sub-districts, of which 45% (485/1,085) were successful (Table 4). Sub-district 1 had 84 referrals (8% of the total referrals), compared to the 1,001 referrals (92% of the total referrals) in Sub-district 2. The success rate in Sub-district 1 was 33% (28/84), compared to the 46% (457/1,001) in Sub-district 2. Table 4 shows the referral success rates according to the referral reasons.

**Table 4.** Referral success rates according to the reason for referral

		Sub-district 1 N (%)	Sub-district 2 N (%)	Total across sub-districts N (%)
Reason for referral				Total (%)^a^
Children < 5 years (e.g. deworming, Vitamin A, immunisation)	Successful referrals	8 (22%)	172 (57%)	180 (53%)
	*Total referrals*	*36*	*303*	*339 (31%)*
Diagnostic tests (being tested or receiving results for all conditions excluding TB/HIV and AIDS)	Successful referrals	2 (100%)	8 (29%)	10 (33%)
	*Total referrals*	*2*	*28*	*30 (3%)*
Finding defaulting clients (including TB/HIV clients)	Successful referrals	1 (50%)	19 (34%)	20 (34%)
	*Total referrals*	*2*	*56*	*58 (5%)*
Male medical circumcision	Successful referrals	0	5 (29%)	5 (29%)
	*Total referrals*	*0*	*17*	*17 (2%)*
Medication collection	Successful referrals	6 (32%)	30 (38%)	36 (37%)
	*Total referrals*	*19*	*79*	*98 (9%)*
Non-communicable disease care	Successful referrals	2 (50%)	5 (28%)	7 (32%)
	*Total referrals*	*4*	*18*	*22 (2%)*
Obstetrics/Gynaecology (including family planning)	Successful referrals	0 (0%)	50 (56%)	50 (56%)
	*Total referrals*	*3*	*90*	*93 (9%)*
Physical symptoms	Successful referrals	2 (33%)	38 (45%)	40 (44%)
	*Total referrals*	*6*	*85*	*91 (8%)*
Reminding clients about facility appointments	Successful referrals	2 (100%)	5 (31%)	7 (39%)
	*Total referrals*	*2*	*16*	*18 (2%)*
TB/HIV/AIDS care	Successful referrals	0	30 (65%)	30 (65%)
	*Total referrals*	*0*	*46*	*46 (4%)*
TB/HIV/AIDS testing	Successful referrals	2 (67%)	60 (43%)	62 (33%)
	*Total referrals*	*3*	*140*	*143 (13%)*
Other^b^	Successful referrals	3 (43%)	35 (28%)	38 (29%)
	*Total referrals*	*7*	*123*	*130 (12%)*
Total	Successful referrals	28 (33%)	457 (46%)	485 (45%)
	*Total referrals*	*84 (8%)*	*1,001 (92%)*	*1,085 (100%*

^a^The % reported for the total of each referral reason is the proportion of the total referrals across sub-districts.

^b^Included a range of health issues, such as wound care, eye care, having to see the occupational therapist or social worker, and mental healthcare. It also included unspecified reasons, when the recall/referral simply stated that the client needed to seek care at the facility.

Data from the implementation journal suggested that one facility in Sub-district 2 had a higher referral success rate than the others because the nursing staff had prioritised LHW referrals. Clients arriving at this facility were, therefore, more closely followed by clerks. This was corroborated by the interview data, where some clerks indicated that certain referrals and recalls were made and closed based on priorities and targets set by the facility – and sub-district management:

*Every month they* [management] *check … if there are reports that have to be signed off … So she* [the clerk] *knew that these had to go out*. (Sub-district 1, Facility 2 clerk)

The interviews suggested several reasons why success rates for recalls and referrals were low. Closing the recalls and referrals was referred to by clerks as ‘*the worst part of the job*’. They described a lack of feedback from the healthcare professionals about who had arrived after a recall or referral, and that receiving feedback from the LHWs about whether a client was found, sometimes lacking, too:

*The feedback wasn’t always there, especially when you sent out something to the home-based carers* [LHWs], *you just don’t get feedback*. (Sub-district 2, Facility 3 clerk)

This lack of feedback from LHWs meant that to close the recalls and referrals, clerks had to either bother busy healthcare professionals; have in-person confirmation from the recalled or referred clients when seeing the client arriving at the facility; or they had to obtain client folders from the rooms in which they were stored. This impacted on their perceptions of the system itself. Similar to referrals, if interpersonal relationships between clerks and LHWs or between clerks and the healthcare professionals were strained, it hampered the closing of recalls. This was described by clerks in three of the facilities:

*In the beginning she* [the nurse responsible for checking folders] *told me the* [recalled] *child had arrived … but then there was …* [an incident]. *Something between me and her … that she wasn’t able to tell me* [when recalled clients arrived]*….* (Sub-district 2, facility 1, clerk)

To support closing recalls and referrals, most clerks suggested developing a recording system at the reception that would enable identifying recalled or referred clients without depending on memory or personal communication. Poor internet and mobile phone connections, and staff absences, also contributed to unclosed recalls and referrals: when only one person was responsible for the system, it did not function when that person was absent.

The clerks’ description of the challenges with closing recalls was corroborated by data showing that only 8% (n = 129) of recalls across the sub-districts failed because clients did not attend the facility as requested (Table 5). The remaining 92% of failures were due to the following issues: 66% of the failures were ‘unclosed recalls’, with the final correspondence in the system coming either from the clerk or LHW, without the other party responding; in 17% of failed recalls, LHWs did not view the recall; and 9% of failures were due to clerk errors, referring to clerks assigning a recall to an LHW not serving the area in which the client lived, or creating a recall without assigning an LHW to it.

**Table 5.** Reasons for failed recalls

	Sub-district 1 N (%)	Sub district 2 N (%)	Total N (%)
Client did not attend the facility	43 (16%)	86 (7%)	129 (8%)
Unclosed recall	161 (62%)	871 (66%)	1033 (66%)
LHWs did not view the recall	51 (20%)	215 (16%)	266 (17%)
Clerk error	6 (2%)	142 (11%)	148 (9%)
Total failed recalls	261	1,314	1,575

### Referral and recall implementation and feasibility

Despite the low success rate for recalls, which the clerks themselves also lamented, clerks and some managers were generally optimistic that the mHealth system had had a positive impact and was feasible to implement:

*WO:* *Did you notice at all that more people were coming* [to the facility] *now than in the past?*

*Manager:* *Yes, definitely … I don’t know if we have the statistics, but all we can say is that the clinic’s headcount has increased, especially at* [Facility 1] … *our chronic service there has improved a lot. And I think the new system is definitely responsible for that.”* (Sub-district 1, Facility 1 manager)

*Yes, like I’ve said, it worked very well. It worked very well because we could send people* [LHWs to recall clients] *out. (Sub-district 1, Facility 3 clerk)*

Only one clerk thought that there was no difference in results between the mHealth and paper systems. She was of the view that only the mechanism had changed, and not the work itself, but even she found the mHealth system more convenient than the paper-based system. Others reported benefits such as increased immediacy in issuing recalls and in getting feedback on these, as well as reaching more clients across a wider geographic area.

*It was nice to work like that because if you sent out a message, then you immediately got a reply from the home-based carer* [LHW]. (Sub-district 1, Facility 2 clerk)

*But with the tablet we could address a wider population, and also on their (LHW) routes they could address more people if they had cell phones because you were able to send messages to them.* (Sub-district 2, Facility 2 clerk)

While most staff acknowledged that the mHealth system was not perfect, they were generally of the view that it was more reliable and functional than the paper-based system. They also saw the mHealth system as offering accountability, as records were retained of when recalls or referrals were made. The clerks and managers also recommended a number of improvements to the system itself, including using data from existing electronic patient records to populate clients’ personal details in the mHealth system.

## Discussion

The mHealth system evaluated in this study aimed to improve continuity of care in rural and semi-rural communities in South Africa. However, the success rates of the intervention modalities were low, at 28% and 45%, respectively, for recalls and referrals.

The qualitative data from our study suggest that while the mHealth system was appreciated by clerks and health professionals at the facilities, there were a number of challenges in implementing the system. The biggest challenge was for clerks to record the outcomes of recalls and referrals. It is, therefore, possible that the true recall and referral success rates were higher than those measured, given that a large proportion of cases (66% across the two sub-districts) were left unclosed by either the clerk or LHW. One approach to overcoming this challenge in future implementations of similar mHealth systems could be to integrate the mHealth functions into facility-level electronic health information systems, where these already exist. For example, a Zambian study similar to ours, but with automated issuing of recalls, achieved a much higher recall success rate (63%) than the 28% in our study [38]. Several studies confirm the need for integration, claiming that it may strengthen health system functioning [39] and promote the sustainability of the mHealth system [40]. As was found in our study, poor integration may frustrate users and lead to additional labour-intensive tasks for them [41,42]. With this in mind, automated entry, as suggested by the clerks, could help to ensure that staff with many other responsibilities do not have to spend time on manually issuing and closing recalls and referrals. In addition, this could lead to more accurate data on success rates.

The feasibility of our system was further challenged by the fact that clerks already had high workloads prior to being tasked with managing the mHealth recalls and referrals. In some facilities, they had no additional support with these tasks, and allocating such support could be difficult in overburdened and understaffed PHC facilities in resource-constraint settings. As in several other studies [42–44], we found that successfully maintaining an mHealth system was a challenge when added to staff’s existing job descriptions. In addition, many clerks reported struggling with the technical aspects of the mHealth system. Given that some clerks had not used a tablet before, this is not surprising. These findings show how health system absorption capacity, in terms of human resources, is key to the success of any mHealth intervention, and provide an important ‘reality check’ to the widespread optimism regarding the impacts of digital health interventions delivered via mobile.

Our results show that success rates and recalls and referrals varied greatly between sub-districts. Direct comparisons between these two settings are difficult, as infrastructure and population size vary greatly. The differences between sub-districts could partly be attributed to facilities, that set priorities based on needs and service performance audits. The qualitative and quantitative data showed that context influenced the number and content of recalls and referrals greatly. This suggests that the system is adaptable to different needs across different settings. Similarly, the success rates for recalls and referrals were strongly influenced by a single, well-performing facility in each sub-district. In the participating hospital in Sub-district 1, the recalls comprised of notifying LHWs when newborns were sent home from the hospital. When LHWs reported that they had contacted the family and had enrolled the child into their care, the clerk closed the recall as successful. Given that this task is much easier than, for example, ensuring that a client who has defaulted from their medication returns to a facility, the hospital recall success rate was much higher (77%) than for any other facility in that sub-district.

The referral and recall process in our study depended on teamwork and communication within the facility, as healthcare professionals needed to share information with the clerk to ensure that referral and recalls were issued and closed appropriately. Therefore, facility-based variation was influenced by interpersonal relationships within the facility, with these relationships being critical to how well a facility functions [45]. Finding recall or referral outcome information from individual client folders was difficult. Another South African project focusing on referrals for tuberculosis case finding [46] gave clients barcodes that allowed referrals to be identified on arrival at the facility. This suggests that to improve the feasibility of interventions such as ours, attention should be paid to completing the feedback loop from facility to community and back to the facility, through a standardised system that relieves pressure from busy professionals. Such approaches may be more complicated and expensive to implement, but may be a useful approach for tracking recalls and referrals more reliably at facilities.

Although this paper does not report on client perspectives regarding the mHealth system (these findings will be reported separately), client-related factors, including how far from the facility they live, and their values and preferences regarding their health, are also likely to impact on the success of recalls and referrals [47]. This makes it difficult to predict how clients will respond to health workers’ encouragement to seek healthcare [48]. Ensuring that clients attend the facility when requested to do so is, therefore, a complex social process in which encouragement plays only a part [49].

Our study suggests that, broadly speaking and taking into account the caveats discussed above, it may be feasible to implement this mHealth system in similar settings, and that it offers advantages, including immediacy and improvement in communication and accountability between LHWs and facility staff. It is also adaptable according to facility needs. Importantly, this mHealth intervention enabled LHWs to receive instructions and report progress while in the community, which could translate to important gains in process timing. These advantages have also been noted in other mHealth studies [9,23,50]. However, the low recall and referral success rates, and the challenges highlighted in the qualitative data, suggest that the implementation of mHealth interventions in the ‘real world’ primary healthcare services is challenging. The capacity of the local health system to implement and sustain such interventions needs to be assessed in advance and monitored closely during implementation.

The system encountered obstacles such as poor internet access, changes in staffing and clerks already burdened with other duties. Further implementation research is needed to address questions such as how mHealth systems can be implemented with minimal disruption to facility staff and service delivery processes, how best to integrate it with existing health information systems and the cost-effectiveness of these approaches. A forthcoming qualitative evidence synthesis will also provide valuable insights regarding the healthcare workers’ perceptions and experiences reported in this paper [51]. This mHealth intervention was implemented within the real world of PHC services in under-resourced, rural and semi-rural communities, with no additional staff employed to manage the mHealth system. This study confirms that it is possible to implement these systems in real-world settings [38]. Based on our evaluation, we present suggestions for similar mHealth interventions in Box 1.

**Box 1.** Suggestions for future mHealth interventions

Integrate mHealth systems with existing health information systems in the implementation context. Support the implementation of mHealth systems with training directed to all facility staff. It may be helpful for this training to cover relevant interpersonal communication skills and communication channels. Explore ways of ensuring buy-in from all implementers. Ensure that mHealth applications do not result in significant increases in workload for staff assigned to manage the mHealth system. Explore ways of providing on-site technical support to teams, for example, through having an online support desk.

Our mixed-methods design provided a more nuanced understanding [52,53] of the implementation successes and challenges of the mHealth system than could have been achieved through one method alone, with the qualitative findings being used to understand and corroborate the quantitative findings [28,29,54]. We enhanced the trustworthiness of our qualitative data through including one of the NGO supervisors and research participants [PH] as co-author. In this way, we were able to confirm that our interpretations of the qualitative data resonated with her and her colleagues’ perceptions and experiences. This was a type of ‘member checking’ [55], which can improve the trustworthiness of qualitative findings.

This study has several limitations: first, the mHealth system was discontinued by the Western Cape Government’s Department of Health after the project funding ended. We, therefore,cannot comment on the sustainability and performance of the system over time. Nevertheless, the project provided insights on sustainability considerations for digital health interventions of this kind, for example, in relation to the need for continuous training of staff and the importance of embedding new interventions into current systems (Box 1). Second, the study was conducted only in two, rather different semi-rural South African communities, and therefore generalisability is limited. Third, due to a lack of integration with existing health information systems as well as resource constraints, we were unable to assess the intervention’s impacts on client’s health, for example, whether better recall led to improvements in blood pressure levels for clients living with hypertension. We were also not able to use the existing health information system to automatically populate personal details in the mHealth system, as the two systems were not linked. Given the absence of data on how successful the paper-based system was in recalling and referring clients and community members, we were unable to make a direct comparison between the paper-based and mHealth systems, beyond participants’ reported experiences. Similarly, we did not have data to allow us to compare the costs of the paper-based system to the monthly mHealth costs per device, which was 3 US$ for technical support from the service provider, 3 US$ for data bundles, and 14 US$ per for platform hosting.

## Conclusions

Mobile health interventions to improve the continuity of primary healthcare are probably feasible to implement in rural and semi-rural communities in poorer settings, but there remain a number of challenges to their implementation. Our study found that health facility staff appreciated aspects of the system but also experienced challenges and asked for modifications, including automated input of client details from the health information system, as well as automated recording when recalled and referred clients and community members arrived at the facility. Future studies should investigate the absorption capacity of health systems to adopt new mHealth interventions as well as whether such systems can improve clients’ health outcomes.

## Paper context

What is already known: mHealth has the potential to improve health outcomes.

What this paper adds: mHealth may improve communication between lay health workers and primary health facility staff, but the success of these programmes depends on the capacity of the health system to incorporate these innovations.

What action needs to be taken: mHealth programmes need to be integrated with existing electronic health information systems and fit into the workload of primary healthcare staff.

## Appendix

**Table A1.** Recall and referral reason categorisation

	Description
**Children < 5**	Checking that immunisation, deworming, and Vitamin A injections were up-to-date, and monitoring their growth and nutrition status
**Diagnostic tests** (excluding TB/HIV/AIDS)	Clients to collect test results or have a blood test done for non-communicable diseases
**Facility appointment reminders**	LHWs reminded clients about appointments or informed them about rescheduled appointments
**Finding defaulting clients**	Clients who have missed facility appointments, or whom the LHWs were concerned that they are not adhering to their treatment
**Male medical circumcision**	Encouraging males to have medical circumcision done; applied to referrals only
**Medication collection**	Reminding clients to collect their medication at the facility
**Non-communicable disease care**	Facility care for clients on hypertension and/or diabetes treatment
**Obstetrics/Gynaecology**	Females were referred or recalled for maternal care, such as papsmear tests, routine antenatal visits, and family planning advice
**Physical symptoms**	People who complained about aches, swollen limbs, skin rash, and similar symptoms; applied to referrals only
**TB/HIV/AIDS care**	Routine check-up visits
**TB/HIV/AIDS testing**	Encouraging community members to be tested for TB and HIV/AIDS; applied to referrals only
**Other**	A variety of issues, including wound care, eye care, having to see the occupational therapist or social worker, and mental healthcare. It also included unspecified reasons, when the recall/referral simply stated that the client needed to seek care at the facility

## References

[1] Adjaye-Gbewonyo K, Kawachi I, Subramanian SV, et al. Income inequality and cardiovascular disease risk factors in a highly unequal country: a fixed-effects analysis from South Africa. Int J Equity Health. 2018;17:31. 29510733 10.1186/s12939-018-0741-0 PMC5839065

[2] Pillay-van Wyk V, Msemburi W, Laubscher R, et al. Mortality trends and differentials in South Africa from 1997 to 2012: second national burden of disease study. Lancet Glob Health. 2016;4:e642–14. 27539806 10.1016/S2214-109X(16)30113-9

[3] Mayosi BM, Flisher AJ, Lalloo UG, et al. The burden of non-communicable diseases in South Africa. Lancet. 2009;374:934–947. 19709736 10.1016/S0140-6736(09)61087-4

[4] Surender R, van Niekerk R, Alfers L. Is South Africa advancing towards national health insurance? The perspectives of general practitioners in one pilot site. South Afr Med J. 2016;106:1092–1095. 10.7196/SAMJ.2016.v106i11.10683 27842630

[5] Schneider H, Besada D, Daviaud E, et al. Ward-based primary health care outreach teams in South Africa: developments, challenges and future directions. South Afr Health Rev. 2018;2018:59–65.

[6] Labonté R, Sanders D, Mathole T, et al. Health worker migration from South Africa: causes, consequences and policy responses. Hum Resour Health. 2015;13:92. 26635007 10.1186/s12960-015-0093-4 PMC4669613

[7] Portoghese I, Galletta M, Coppola RC, et al. Workload among health care workers: the moderating role of job control. Saf Health Work. 2014;5:152–157. 25379330 10.1016/j.shaw.2014.05.004 PMC4213899

[8] van Ginneken N, Lewin S, Berridge V. The emergence of community health worker programmes in the late apartheid era in South Africa: an historical analysis. Soc Sci Med. 2010;71:1110–1118. Epub 2010/ 07/20. PubMed PMID: 20638169; PubMed Central PMCID: PMCPmc2941026. 20638169 10.1016/j.socscimed.2010.06.009 PMC2941026

[9] Schneider H. The challenges of reshaping disease specific and care oriented community based services towards comprehensive goals: a situation appraisal in the Western Cape Province, South Africa. BMC Health Serv Res. 2015;15:1–11. 26424509 10.1186/s12913-015-1109-4 PMC4589097

[10] Atkins S. Lay health worker-supported tuberculosis treatment adherence in South Africa: an interrupted time-series study. Int J Tuberc Lung D. 2011;15:84–89. 21276302

[11] Odendaal W. Multiple and mixed methods in formative evaluation: is more better? reflections from a South African study. BMC Med Res Methodol. 2016;16:173. 27978818 10.1186/s12874-016-0273-5 PMC5159984

[12] Nkonki L CJ, Sanders D. Lay health worker attrition: important but often ignored. Bull World Health Organ. 2011;89:919–923. 22271950 10.2471/BLT.11.087825 PMC3260896

[13] Castro Lopes S, Guerra-Arias M, Buchan J, et al. A rapid review of the rate of attrition from the health workforce. Hum Resour Health. 2017;15:21. 28249619 10.1186/s12960-017-0195-2 PMC5333422

[14] Brunie A, Wamala-Mucheri P, Otterness C, et al. Keeping community health workers in Uganda motivated: key challenges, facilitators, and preferred program inputs. Glob Health Sci Pract. 2014;2:103–116. Epub 2014/ 10/03. PubMed PMID: 25276566; PubMed Central PMCID: PMCPmc4168609. 25276566 10.9745/GHSP-D-13-00140 PMC4168609

[15] Glenton C. Barriers and facilitators to the implementation of lay health worker programmes to improve access to maternal and child health: qualitative evidence synthesis. Cochrane Database Syst Rev. 2013;10. DOI:10.1002/14651858.CD010414.pub2 PMC6396344 24101553

[16] Odendaal W. The provision of TB and HIV/AIDS treatment support by lay health workers in South Africa: a time-and-motion study. Hum Resour Health. 2014;12:1–7. 24708871 10.1186/1478-4491-12-18 PMC3978134

[17] Chang LW. Impact of a mHealth intervention for peer health workers on aids care in rural Uganda: a mixed methods evaluation of a cluster-randomized trial. AIDS Behav. 2011;15:1776–1784. 21739286 10.1007/s10461-011-9995-x PMC3265752

[18] WHO. Global diffusion of eHealth: making universal health coverage achievable. Report of the third global survey on eHealth Report. 2016.

[19] Aranda-Jan CB. Systematic review on what works, what does not work and why of implementation of mobile health (mHealth) projects in Africa. BMC Public Health. 2014;14:188. 24555733 10.1186/1471-2458-14-188 PMC3942265

[20] Madon S. Can mobile phones help control neglected tropical diseases? Experiences from Tanzania. Soc Sci Med. 2014;102:103–110. 24565147 10.1016/j.socscimed.2013.11.036

[21] Braun R. Community health workers and mobile technology: a systematic review of the literature. PLoS One. 2013;8. DOI:10.1371/journal.pone.0065772. PMC3680423 23776544

[22] Henry J. Enhancing the supervision of community health workers with whatsapp mobile messaging: qualitative findings from 2 low-resource settings in Kenya. Global Health. 2016; DOI:10.9745/GHSP-D-15-00386 PMC4982254 27353623

[23] Mangwi-Ayiasi R. Use of mobile phone consultations during home visits by community health workers for maternal and newborn care: community experiences from Masindi and Kiryandongo districts, Uganda. BMC Health Serv Res. 2015;15:560. 26084369 10.1186/s12889-015-1939-3 PMC4471930

[24] Praveen D, Patel A, Raghu A, et al. SMARTHealth India: development and field evaluation of a mobile clinical decision support system for cardiovascular diseases in rural India. JMIR Mhealth Uhealth. 2014;2:e54.PubMed PMID: 25487047; PubMed Central PMCID: PMC4275493 25487047 10.2196/mhealth.3568 PMC4275493

[25] Hirsch-Moverman Y, Daftary A, Yuengling KA, et al. Using mHealth for HIV/TB treatment support in Lesotho: enhancing patient–provider communication in the START study. J Acquir Immune Defic Syndr. 2017;74:S37–S43.PubMed PMID: PMC5147041 27930610 10.1097/QAI.0000000000001202 PMC5147041

[26] Jennings L, Ong’ech J, Simiyu R, et al. Exploring the use of mobile phone technology for the enhancement of the prevention of mother-to-child transmission of HIV program in Nyanza, Kenya: a qualitative study. BMC Public Health. 2013;13:1131. 24308409 10.1186/1471-2458-13-1131 PMC4234194

[27] Feilzer YM. Doing mixed methods research pragmatically: implications for the rediscovery of pragmatism as a research paradigm. J Mixed Methods Res. 2009;4:6–16.

[28] Bloch C, Sorensen MP, Graversen EK, et al. Developing a methodology to assess the impact of research grant funding: a mixed methods approach. Eval Program Plann. 2014;43:105–117. Epub 2014/ 01/15. PubMed PMID: 24418571. 24418571 10.1016/j.evalprogplan.2013.12.005

[29] Odendaal W, Atkins S, Lewin S. Multiple and mixed methods in formative evaluation: is more better? reflections from a South African study. BMC Med Res Methodol. 2016;16:173. 27978818 10.1186/s12874-016-0273-5 PMC5159984

[30] Creswell JW, Plano Clark VL. Choosing a mixed methods design. In: Creswell JW, Plano Clark VL, editors. Designing and conducting mixed methods research. Thousand Oaks, CA: SAGE Publications; 2007. p. 62–79.

[31] Government WC. Eden: population size, poverty and health. Report. 2016.

[32] District E. Sub-district 1: facility headcount – 2015–16. Report. 2016.

[33] District E. Sub-district 2: facility headcount – 2015–16. 2016.

[34] Government WC. Eden report: population demographics. Report. 2013.

[35] Schneider H, Nxumalo N. Leadership and governance of community health worker programmes at scale: a cross case analysis of provincial implementation in South Africa. Int J Equity Health. 2017;16:72. 28911324 10.1186/s12939-017-0565-3 PMC5599898

[36] WHO. Classification of digital health interventions v1.0. Geneva: World Health Organisation; 2018. p. 1–20.

[37] Graneheim UH, Lundman B. Qualitative content analysis in nursing research: concepts, procedures and measures to achieve trustworthiness. Nurse Educ Today. 2004;24:105–112. Epub 2004/ 02/11. PubMed PMID: 14769454. 14769454 10.1016/j.nedt.2003.10.001

[38] Schuttner L, Sindano N, Theis M, et al. A mobile phone-based, community health worker program for referral, follow-up, and service outreach in rural Zambia: outcomes and overview. Telemed J E Health. 2014;20:721–728.PubMed PMID: PMC4106387 24926815 10.1089/tmj.2013.0240 PMC4106387

[39] Labrique AB, Vasudevan L, Kochi E, et al. mHealth innovations as health system strengthening tools: 12 common applications and a visual framework. Global Health. 2013;1:160. 10.9745/GHSP-D-13-00031 PMC4168567 25276529

[40] Lodhia V, Karanja S. Acceptability, usability, and views on deployment of peek, a mobile phone mhealth intervention for eye care in Kenya: qualitative study. JMIR Mhealth Uhealth. 2016;4:e30. PubMed PMID: 27160779. 10.2196/mhealth.4746 PMC4877502 27160779

[41] Garg SK, Lyles CR, Ackerman S, et al. Qualitative analysis of programmatic initiatives to text patients with mobile devices in resource-limited health systems. BMC Med Inform Decis Mak. 2016;16:16. Epub 2016/02/08. PubMed PMID: 26851941; PubMed Central PMCID: PMCPmc4744448. 26851941 10.1186/s12911-016-0258-7 PMC4744448

[42] Rothstein JD, Jennings L, Moorthy A, et al. Qualitative assessment of the feasibility, usability, and acceptability of a mobile client data app for community-based maternal, neonatal, and child care in rural Ghana. Int J Telemed Appl. 2016;2016:2515420. Epub 2017/ 01/11. PubMed PMID: 28070186; PubMed Central PMCID: PMCPMC5192299 publication of this paper. 28070186 10.1155/2016/2515420 PMC5192299

[43] Wolff-Piggott B, Coleman J, Rivett U. The clinic-level perspective on mHealth implementation: a South African case study. Inf Technol Dev. 2018;24:532–553.

[44] Kolltveit B-CH, Gjengedal E, Graue M, et al. Conditions for success in introducing telemedicine in diabetes foot care: a qualitative inquiry. BMC Nurs. 2017;16:2. 28100957 10.1186/s12912-017-0201-y PMC5237197

[45] Jooste K, Hamani M. The motivational needs of primary health care nurses to acquire power as leaders in a mine clinic setting. Health SA. 2017;22. DOI:10.4102/hsag.v22i0.961

[46] Ayles H, Muyoyeta M, Du Toit E, et al. Effect of household and community interventions on the burden of tuberculosis in southern Africa: the ZAMSTAR community-randomised trial. Lancet. 2013;382:1183–1194. Epub 2013/08/07. PubMed PMID: 23915882. 23915882 10.1016/S0140-6736(13)61131-9

[47] Ames HM, Glenton C, Lewin S, et al. Clients’ perceptions and experiences of targeted digital communication accessible via mobile devices for reproductive, maternal, newborn, child, and adolescent health: a qualitative evidence synthesis. Cochrane Database Syst Rev. 2019;10:CD013447–CD. PubMed PMID: 31608981 31608981 10.1002/14651858.CD013447 PMC6791116

[48] Ellis J, Boger E, Latter S, et al. Conceptualisation of the ‘good’ self-manager: a qualitative investigation of stakeholder views on the self-management of long-term health conditions. Soc Sci Med. 2017;176:25–33. 28126586 10.1016/j.socscimed.2017.01.018

[49] Nutting PA, Goodwin MA, Flocke SA, et al. Continuity of primary care: to whom does it matter and when? Ann Fam Med. 2003;1:149–155. Epub 2004/ 03/27.PubMed PMID: 15043376; PubMed Central PMCID: PMCPmc1466596 15043376 10.1370/afm.63 PMC1466596

[50] Medhanyie AA, Moser A, Spigt M, et al. Mobile health data collection at primary health care in Ethiopia: a feasible challenge. J Clin Epidemiol. 2015;68:80–86.PubMed PMID: 25441699 25441699 10.1016/j.jclinepi.2014.09.006

[51] Odendaal W, Goudge J, Griffiths F, et al. Healthcare workers’ perceptions and experiences on using mHealth technologies to deliver primary healthcare services: a qualitative evidence synthesis. Cochrane Database Syst Rev. 2015;2015. PubMed PMID: 27478408; PubMed Central PMCID: PMCPmc4966615. Epub 2016/ 08/02. DOI:10.1002/14651858.cd011942 PMC4966615 27478408

[52] Betzner A, Lawrenz FP, Thao M. Examining mixing methods in an evaluation of a smoking cessation program. Eval Program Plann. 2016;54:94–101. Epub 2015/ 11/02. PubMed PMID: 26520457. 26520457 10.1016/j.evalprogplan.2015.06.004

[53] Meschede T, Chaganti S. Home for now: a mixed-methods evaluation of a short-term housing support program for homeless families. Eval Program Plann. 2015;52:85–95. Epub 2015/ 05/20. PubMed PMID: 25989204. 25989204 10.1016/j.evalprogplan.2015.03.009

[54] Denzin N. Strategies of multiple triangulation. In: The research act: a theoretical introduction. 3rd ed. New Jersey: Prentice-Hall; 1989. p. 234–247.

[55] Birt L, Scott S, Cavers D, et al. Member checking: a tool to enhance trustworthiness or merely a nod to validation? Qual Health Res. 2016;26:1802–1811. PubMed PMID: 27340178 27340178 10.1177/1049732316654870

